# Vertebral Body Tethering for Thoracolumbar Curvatures in Adolescent Idiopathic Scoliosis: Radiographic and Clinical Outcomes at 2–6-Year Follow-Up

**DOI:** 10.3390/jcm13216330

**Published:** 2024-10-23

**Authors:** Lily Eaker, Olgerta Mucollari, Noor Maza, Baron Lonner

**Affiliations:** Department of Orthopaedic Surgery, Mount Sinai Hospital, New York, NY 10029, USA; lily.eaker@gmail.com (L.E.); olgerta.mucollari@mountsinai.org (O.M.); noor.maza@mountsinai.org (N.M.)

**Keywords:** vertebral body tethering, adolescent idiopathic scoliosis, posterior spinal fusion, thoracolumbar curvature

## Abstract

**Background:** The gold standard treatment for adolescent idiopathic scoliosis (AIS) is posterior spinal fusion (PSF). However, long-term consequences of PSF can include reduced spinal flexibility, back pain, and intervertebral disc degeneration. Vertebral body tethering (VBT) is a non-fusion alternative that preserves motion. We investigated the outcomes of VBT for the treatment of thoracolumbar (TL) major AIS in the largest single-surgeon series with a minimum 2-year follow-up (FU). **Methods:** We performed a retrospective single-center review. Inclusion criteria were AIS, Lenke 5/6 curvature, and skeletally immature Variables were compared using Student’s *t*-tests, Wilcoxon rank sum tests, Chi-square, and Fisher’s exact tests. **Results:** A total of 37 consecutive patients, age 14.1 ± 1.6 years, 86.5% F, 35.9 ± 11.5-month FU, were examined. Overall, 27 patients (73%) had Lenke 5 and 10 (27%) had Lenke 6 curvatures. Instrumentation of the TL curve alone was performed in 59.5%, and thoracic (T) and TL in 40.5% of patients. Overall, 45.9% of patients had two tethers placed in the TL spine; no patients had double tethers placed at the main thoracic curves. The TL (51 ± 8° to 20 ± 11°; *p* < 0.0001) and T (37 ± 13° to 17 ± 10°; *p* < 0.0001) curvatures improved from baseline to the latest FU. Overall, 89% of patients achieved major Cobb < 35°; the three patients who did not experienced at least one cord breakage or required PSF. T5-T12 kyphosis increased (*p* = 0.0401) and lumbar lordosis was maintained (*p* = 0.9236). Both the TL inclinometer (16 ± 4º to 4 ± 2°; *p* < 0.0001) and T (6 ± 4° to 4 ± 3°; *p* = 0.0036) measurements improved. There was a 49% tether breakage rate as follows: 60% for single-cord TL constructs and 35% for double cords (*p* = 0.0991). There was an 8.1% re-operation rate as follows: one conversion to T PSF and revision of the TL tether; one release of the T tether and revision of the TL tether; one screw revision for radiculopathy. One patient was re-admitted for poor pain control. **Conclusions:** Patients with TL major curvature treated with VBT experienced a high rate of clinically successful outcomes with maintenance of lumbar lordosis and relatively low complication rates at the latest FU.

## 1. Introduction

Vertebral body tethering (VBT) is a promising non-fusion alternative to the current gold-standard posterior spinal fusion (PSF) for the treatment of adolescent idiopathic scoliosis (AIS) [1,2,3]. Current VBT studies have shown variability in outcomes impacted by patient selection; however, most are focused on VBT for the main thoracic curvatures. Of those inclusive of patients treated with thoracolumbar VBT, studies have been limited by shorter follow-up or a mixture of treated curve types [2,4,5]. PSF, particularly into the lumbar spine, can be associated with long-term sequelae [6,7,8,9,10]. In addition to growth preservation, benefits of VBT include maintenance of at least some motion within instrumented segments, improved trunk endurance and the potential for decreased incidence of disc degeneration compared to PSF [11,12,13]. VBT may have the greatest clinical benefit for those who would otherwise require lumbar fusions. 

However, while VBT holds promise for lumbar curvatures, there is concern about the unknown long-term outcomes, particularly regarding tether cord durability [4,14]. Tether breakage rates following thoracic VBT have been as high as 48%, with many suspecting tether breakage rates to be higher in lumbar tethers [1,4,14,15]. We sought to study the clinical and radiographic outcomes of VBT in patients with thoracolumbar/lumbar major curvatures. 

## 2. Materials and Methods

Institutional review board approval was obtained for this single-surgeon retrospective review of VBT patients. For this study, the operating surgeon did not perform radiographic measurements or statistical analysis, which was instead performed by the research coordinator. Skeletally immature patients (Risser ≤ 4) with AIS, Lenke 5/6 curvature, and a minimum 2-year follow-up (FU) were included. In total, 53 patients met the criteria, but 16 were excluded [12 had ≤2-year FU (9.8 ± 5.4 months); 4 were lost to FU]. Of the 12, all patients had thoracic and thoracolumbar Cobb angles below 30° with no re-operations. In total, 2 patients experienced complications: 1 superficial wound infection requiring oral antibiotics, which resolved without sequelae, and 1 incidence of incontinence thought to be related to urinary tract infection. Indications for VBT included thoracolumbar curvatures ≤75° that were flexible to ≤35° on bending X-rays. The secondary thoracic curvature was included if the thoracic curvature was structural according to Lenke criteria, with a curvature of ≥40°, wedging of apical vertebrae, clinical rib prominence (≥10°) and/or pain. Patients who had surpassed their peak height velocity were advised of the controversial nature of this indication. The surgeon offered VBT to patients at more advanced stages of maturity due to the potential drawbacks of fusion into the lumbar spine [16]. 

The procedure is performed via a standard thoracoabdominal exposure between the 10th and 11th ribs and retroperitoneal dissection to access T11 to L4. The diaphragm is incised at its periphery, leaving a 1.5 cm cuff along the chest wall and spine for lateral repair. T10 is easily accessed via a portal placed in the intercostal space above the 10th or 9th rib placed within a subcutaneous flap from the main incision. The incision is typically 10–15 cm. Single-lung ventilation with the use of a bronchial blocker is performed to facilitate easy access to the lower thoracic spine without the impediment of a ventilated lung during surgery. The psoas muscle is disserted and retracted posteriorly. Proximal levels up to T9 or more proximally can be accessed via a 15 mm posterior axillary line portal. After completion of the procedure, a chest tube is placed through a 5 mm anterior axillary line portal site used for the placement of a scope that provides visualization and lighting for the procedure, the lung is reinflated, and the incisions are closed in layers. The levels selected for instrumentation are typically inclusive of Cobb end vertebrae (CEV) and extend one level caudal to the distal CEV in cases in which the CEV is not touched by the central sacral vertical line. Early in the series, a single tether and row of screws were routinely utilized for the thoracolumbar curvature, whereas later, a double row of screws and second tether, at least from T11/T12 to L3/L4, were utilized for curves >60°, Risser 3, and/or flexibility <50%.

### 2.1. Data Collection

Clinical and radiographic parameters were collected at baseline, 1st erect, and latest FU (≥2-year FU). Hand radiographs were not routinely available and are not reported here as a result. The proximal humerus ossification system (PHOS) was used to assess skeletal maturity from standard scoliosis PA radiographs in addition to Risser staging [17,18]. Fulcrum bending radiographs were performed to assess curve flexibility. Coronal plane radiographic parameters (proximal thoracic, thoracic, and lumbar curvatures) and sagittal parameters (T5–T12 kyphosis and lumbar lordosis) were assessed along with complications. Tether breakage was defined as an increased of ≥5° in coronal angulation between adjacent screws from 1st erect and latest FU [15]. Clinical success was defined as a major curve <35° without conversion to PSF as previously described [15,19,20]. 

### 2.2. Statistical Analysis 

Descriptive statistics were calculated for all variables. Normality was assessed with Shapiro–Wilk testing. Continuous variables were assessed using Wilcoxon rank sum tests or Student’s *t*-tests, and categorical variables were assessed using Chi-square and Fisher’s exact tests. Significance was set at *p* ≤ 0.05. 

## 3. Results

### 3.1. Baseline Demographics

**Figure 1:**
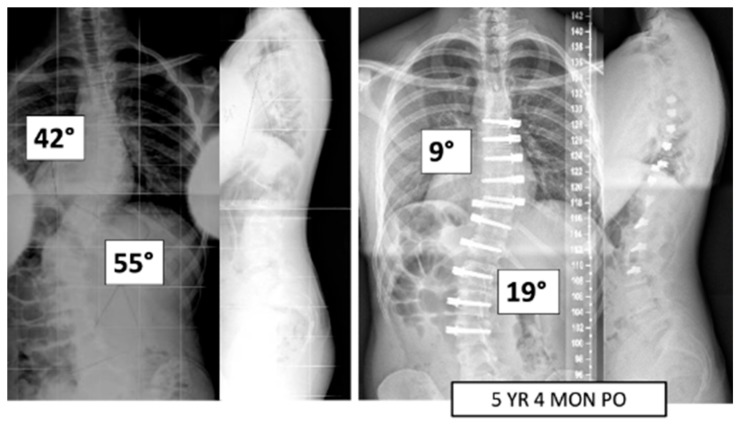
This is a 15-year-old male treated with bilateral VBT instrumentation. At 5 years and 4 months post operation, he has returned to all activities.

**Figure 2:**
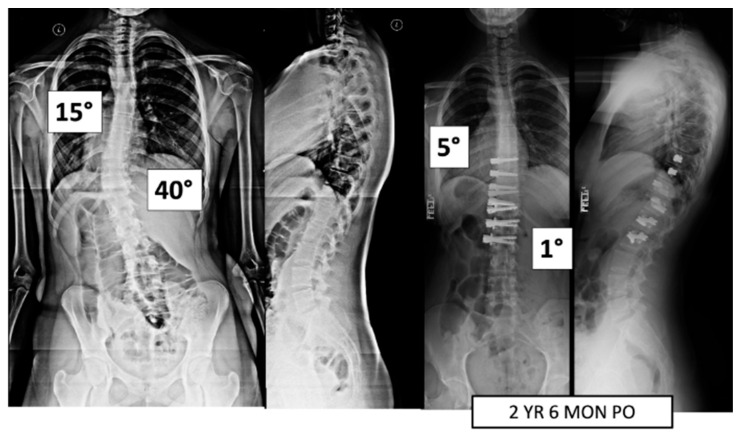
This is a 15-year-old female treated with lumbar VBT. At 2 years and 6 months post operation, she has resumed all activities with no complications.

A total of 37 patients, age 14.1 ± 1.6 years, 86.5% female (Table 1), were included. Pre-operative major Cobb was 51 ± 8° and minor Cobb was 37 ± 13° (Figure 1 and Figure 2). Of the 27 Lenke 5 patients, 6 (22%) had their thoracic curve instrumented in addition to their thoracolumbar curvature. Of the 10 Lenke 6 patients, 9 (90%) had their thoracic curve instrumented as well. The mean FU was 35.9 ± 11.5 months.

### 3.2. Perioperative Characteristics

For those undergoing thoracolumbar instrumentation only, 11 patients (50%) had a single lumbar cord and 11 (50%) had double lumbar cords. Overall, 21 (95%) were Lenke 5 patients and 1 (5%) was a Lenke 6 patient (Table 2). Comparatively, for those with bilateral instrumentation, nine patients (60%) had a single lumbar cord and six (40%) had a double. Six (40%) were Lenke 5 patients and nine (60%) were Lenke 6 patients. In the entire cohort, 17 patients had double cords in the lumbar region. 

### 3.3. Radiographic and Inclinometer Outcomes 

For those with only the major thoracolumbar curvature instrumented (*n* = 22), there was a significant improvement in both major curve (48 ± 6° to 18 ± 12° [62% ± 27%]; <0.0001) and compensatory thoracic curve (29 ± 8° to 15 ± 11° [52 ± 42%]; *p* < 0.0001) between baseline and latest FU (Table 3). There was a slight but non-statistically significant improvement in thoracic T5–T12 kyphosis (20 ± 10° to 24 ± 14°; *p* = 0.323). 

Lumbar lordosis was maintained (53 ± 13 to 52 ± 12; *p* = 0.4664). Thoracolumbar inclinometer measurements improved significantly (16 ± 4° to 5 ± 2°; *p* < 0.0001), but there was no change in the compensatory thoracic inclinometer measurements (4 ± 3° to 3 ± 3°; *p* = 0.4063). T1–S1 height increased between baseline and latest FU (41.2 ± 2.9 cm to 44.4 ± 2.7 cm; *p* = 0.0008). Overall, 20 patients (91%) were considered clinically successful at final FU. A total of 19 patients (86%) had major curve< 30°. Two patients (9%) were considered unsuccessful with major curves >35°. One patient required a revision procedure as described below in the section detailing complications. 

For those with both curvatures instrumented (n = 15), there was a significant improvement in both the major curve (56 ± 8° to 22 ± 9° [60 ± 17%]; *p* < 0.0001) and compensatory thoracic curve (49 ± 8° to 20 ± 7° [59 ± 15%]; *p* < 0.0001) between baseline and latest FU (Table 4). T5-T12 kyphosis improved (26 ± 12° to 32 ± 17°; *p* = 0.0441) and lumbar lordosis was maintained (60 ± 14° to 59 ± 15°; *p* = 0.6435). There were significant improvements in thoracolumbar (17 ± 4° to 4 ± 2°; *p* < 0.0001) and thoracic (9 ± 4° to 5 ± 2°; *p* = 0.0078) inclinometer measurements. T1–S1 height increased between baseline and latest FU (40.3 ± 3.6 cm to 44.4 ± 3.4 cm; *p* = 0.0002). A total of 14 patients (93%) were considered clinically successful at latest FU, and 13 patients (87%) had major curves <30°. One patient (7%) was considered clinically unsuccessful; they experienced radiculopathy caused by an impinging screw tip as described below in the section detailing complications. 

All but one Lenke 6 patient had both T and TL curves instrumented. In the one patient, we elected to treat only the TL curvature as the T curvature was only 40 degrees, was flexible to 26 degrees, and had a small inclinometer measurement of 7 degrees compared to a TL curve magnitude of 53 degrees with an inclinometer of 21 degrees. At final follow-up, the T curvature was 24 degrees, the T inclinometer was 4 degrees, and the TL curve measured 19 degrees with the inclinometer measuring 3 degrees.

Patients with instrumentation of the major curve only were compared to those with bilateral instrumentation. At baseline, those with bilateral instrumentation had significantly larger major curves (56 ± 8° vs. 48 ± 6°; *p* = 0.0011), and compensatory thoracic (49 ± 8° vs. 29 ± 8°; *p* < 0.0001) and proximal thoracic (16 ± 6° vs. 8 ± 5°; *p* = 0.0002) curvatures. While the thoracolumbar inclinometer measurements were comparable at baseline (17 ± 4° vs. 16 ± 4°; *p* = 0.5645), the thoracic measurements (9 ± 4° vs. 4 ± 3°; *p* = 0.0007) were significantly larger in the bilateral instrumentation group. At latest FU, there was comparable correction between the groups for the major curvature (22 ± 9° [60 ± 17%] vs. 18 ± 12° [62 ± 27%]; *p* = 0.7688). There was greater improvement of both the compensatory thoracic (20 ± 7° [59 ± 15%] vs. 15 ± 11° [52 ± 42%]; *p* = 0.0153) and proximal thoracic curvatures (11 ± 7° [32 ± 44%] vs. 8 ± 6° [11 ± 93%]; *p* = 0.0413) in the bilateral instrumentation group. Patients in both groups also had comparable thoracolumbar (4 ± 2° vs. 5 ± 2°; *p* = 0.4054) and thoracic (5 ± 2° vs. 3 ± 3°; *p* = 0.0787) inclinometer measurements, respectively. There was no statistically significant difference between groups in the change in T1 to S1 height between first erect and latest FU (1.5 ± 1.5 cm vs. 2.0 ± 2.1; *p* = 0.5211), respectively. 

At final follow-up, four patients (10.8%) had thoracic hypokyphosis of less than 10 degrees. All four patients had an improvement of kyphosis by an average of 5.3 degrees. The average pre-operative kyphosis for these four patients was 1.8 degrees and increased to 6 degrees. Of these patients, two (50%) had both T and TL curves instrumented, demonstrating subtle T alignment changes with or without instrumentation of the T spine. 

Three patients (8.1%) had T hyper-kyphosis at the final follow-up. Two out of the three patients had pre-operative hyperkyphosis of 45 degrees and 41 degrees, respectively. The third patient had pre-operative kyphosis of 35 degrees. All three had both T and TL curves instrumented. The mean post-operative hyperkyphosis of 46.7 degrees was harmonious with the patients’ pelvic incidence (PI), which averaged 55.3 degrees.

### 3.4. Complications

**Figure 3:**
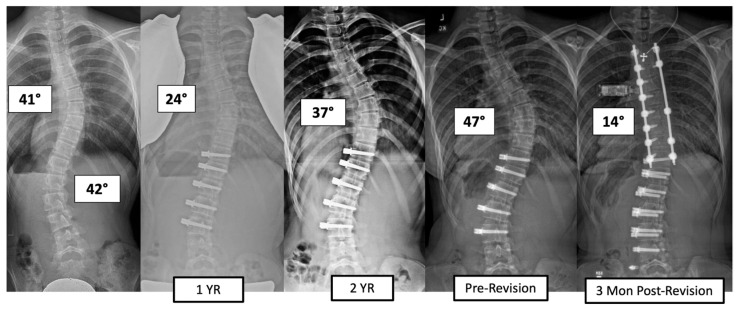
This is a 13-year-old female who experienced progression of the thoracic curve following tether breakage in the lumbar spine. The patient was revised with fusion of the previously untreated thoracic curvature and addition of a second row of screws and second tether for the thoracolumbar construct.

**Figure 4:**
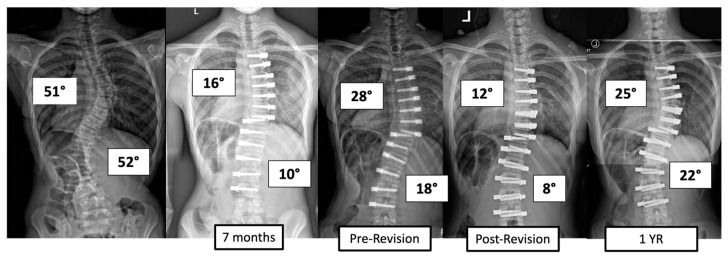
This is an 11-year-old female who experienced overcorrection of the thoracolumbar curvature and adding on of the thoracic. A revision operation was performed to release the tether in the thoracic region and revise the thoracolumbar tether.

There were four major complications (10.8%), including one readmission and three patients who required re-operation. Patient 1 underwent re-operation due to radiculopathy caused by a screw tip impinging on a nerve root in a lumbar foramen requiring screw revision and replacement of the tether. At 3 years post operation, they have a broken tether at one level, but no further revision is needed. Patient 2 had progression of a previously uninstrumented thoracic minor curve which progressed following lumbar tether breakage. This was revised with fusion of the previously untreated thoracic curvature and the addition of a second row of screws and second tether for the thoracolumbar construct (Figure 3). Patient 3 had overcorrection of the thoracolumbar curvature and adding on of the thoracic. As a result, the tether was released in the thoracic region and the thoracolumbar tether was revised (Figure 4). One patient (3%) required readmission due to an inability to tolerate post-operative pain medication. During admission, pain was controlled, and they were discharged without sequela. 

Overall, 18 patients (49%) experienced tether breakage (TB) at latest FU. Of those 18, 13 patients (72%) had a breakage at one level and 5 (28%) at two or more levels. Of the 18, 5 (28%) had bilateral instrumentation, and 5 (28%) had double cords in the thoracolumbar region in which the breakage occurred. There was a 35% TB rate in those with double cords in the lumbar region versus a 60% rate in those with a single cord (*p* = 0.0991). Further, there was a 40% (6 of 15 patients) TB rate in those with bilateral instrumentation versus a 55% (12 of 22 patients) rate in those with only the major curvature instrumented (*p* = 0.5077). 

Overall, 15 of the 18 patients (83%) with TB were still considered clinically successful (major curve <35°) at the time of latest follow-up, and 13 patients (72%) had major curves <30°. 

## 4. Discussion

Non-fusion surgical options and, specifically, VBT for AIS hold the most promise for treatment of the lumbar spine [7,21,22]. Our data provide support for the use of VBT for the treatment of major thoracolumbar curvatures. At 2–6 years follow-up, we found a clinical success rate of 89% (Major Cobb < 35°; all Cobb < 35°) with a complication rate of 10.8%. 

Fusion into the lumbar spine is associated with higher incidence of back pain, decreased mobility within instrumented segments, increased incidence of disc degeneration, and reduced ability to return to sports [7,8,10,11,21]. The standard approach to Lenke 6 curvatures has been the fusion of both curves with some exceptions, in which a selective fusion of the TL major curve is performed. In our study, all but one Lenke 6 patient had both T and TL curves instrumented with a tether, described in our results in greater detail. One could also consider a hybrid posterior fusion of the thoracic curve and tether below, but we have offered that approach only in the setting of severe and rigid thoracic curves and more moderate lumbar curves with a desire to avoid fusion into the lumbar spine. Patients have also been offered a hybrid fusion of the T curve and VBT of the TL curve as an alternative approach more recently, in order to offer the advantages of durability of fusion and flexibility and the potential for less adjacent degenerative disc disease following VBT in TL compared to fusion. Some families have elected for that approach recently. The present study aims to assess the results of VBT only in this case series. 

While a definitive answer to whether vertebral motion segment health will be preserved following VBT is still pending, preliminary MRI studies of the disc and facet health in instrumented and non-instrumented segments of the tethered spine have shown no evidence of degenerative changes to date [13,22]. For our study, the levels instrumented were typically Cobb end vertebrae (CEV) to CEV; however, for the TL curve, tethering was extended to L4 as experience was gained in cases in which L3 was significantly translated and/or rotated, as we have found that we are better able to correct the scoliosis and coronal translation. General guidelines that we have utilized are to include the distal CEV and extend one level distally if the CEV is translated greater than 1.5 cm from CSVL and/or has a Nash–Moe rotation of two or more. In clinical presentations in which the T curve is relatively moderate and flexible and not associated with marked rotational deformity, the surgeon may elect to treat the TL curvature alone as opposed to treating both curves in a Lenke 6 curve type, similar to decision making utilized in fusion indications.

Currently, the only previous study of VBT specifically for lumbar curvatures was conducted by Trobisch et al., who reported on 30 patients with 1 year FU and found a 70% success rate (curve < 30°) with 73% of patients experiencing a tether breakage (TB) [4]. We found that 84% of patients had a final major curve <30° without revision to spinal fusion in the lumbar spine despite a 49% TB rate. While out of the scope of this study, we observed no significant change in sagittal alignment in either the lumbar spine or thoracic spine with either tether breakage or intact tether over time. 

The incidence of TB in the literature has ranged widely (27–73%) and may be even higher as we typically rely on radiographic evidence of breakage [3,4,14,15]. Our TB rate of 49% is consistent with what has been reported in the thoracic VBT literature but lower than what has been reported for lumbar curvatures [3,4]. Baroncini et al. performed an analysis of 105 patients (69 lumbar curves; 84 thoracic curves) who experienced early TB (within 1 year of the index procedure). Of those who experienced breakage, there was a higher incidence amongst lumbar curves (71%) than thoracic (29%) [23]. They suspected that this was due to either the greater range of motion in the lumbar spine or differences in orientation of the facet joints between the thoracic and lumbar spine. In a study on the Lenke 6 curve type, there has been support for bilateral VBT in achieving a significant correction of T and TL curves at a minimum of 24 months post operation [24]. Although TB and loss of correction have been observed, breakage while associated with a segmental loss of correction does not necessarily require re-operation or result in a lack of clinical success (major curve <35%) [1,4,14]. Of the 18 patients in our cohort who experienced TB, 15 (83%) were still clinically successful. Previously, we found a 30% breakage rate of thoracolumbar tethers at the 2-year time point, suggesting that rates increase over time given our 49% rate at 3–6 years [14]. Tether breakage does not appear to be associated with a high revision rate, at least out to the length of the follow-up we report. Of the patients who have been revised in this series (8.1% rate), only one patient who had tether breakage required a revision related to curve progression. This was in a patient in whom a TL tether was performed, breakage occurred, and moderate progression of the TL major curve occurred along with progression of the secondary thoracic curve. This was treated with posterior fusion of the thoracic spine and revision of the TL tether (Figure 3). The interplay between the number of cords and skeletal maturity in outcome durability will require larger data sets. The authors have tended to use single-cord constructs in the lumbar spine when the patient had not yet reached peak height velocity with the expectation that growth modulation would drive correction and maintenance, and a double cord might lead to overcorrection due to a more powerful convex tethering force [25]. The authors routinely use double-row cords for TL major curves for patients who have surpassed peak height velocity and/or patients with curves >60 degrees, regardless of skeletal maturity. 

Our 8% re-operation rate is lower than the 21–41% reported by others for cohorts predominately involving the thoracic spine [1,15,26,27]. This is likely partially due to the inclusion of 70.2% Risser 3–4 and 72.9% PHOS 3B and 4 patients [4,20,28]. Per Li et al., the PHOS 3A stage occurs prior to peak height velocity, whereas PHOS stages 3B and 4 both occur past it when the chance of overcorrection is likely greater [29,30]. Risser staging has been found to be a helpful predictor of the cessation of growth; however, it has been variable in predicting peak height velocity. Little et al. found that peak velocity occurs primarily in the Risser 0 stage, with some experiencing it during Risser 1 [31]. Based upon both skeletal maturity metrics, our cohort had a reduced risk of overcorrection. In a study stratifying VBT outcomes by Sanders stage, Alanay et al. found the highest risk of overcorrection and mechanical complications in Sanders 2 patients versus later Sanders stages [19,28]. Our revision rate is consistent with the only previous clinical series on lumbar VBT, which found a 10% re-operation rate [4]. These early results suggest that the higher breakage rate is not associated with a higher re-operation rate. The use of double-corded constructs may increase the time to breakage. Whether or not holding correction for longer results in better maintenance of correction due to soft tissue and vertebral remodeling is unknown. Whether or not growth modulation has occurred in our patients also requires further study of the wedge angles of apical vertebra. Overall, we did not witness improvement in the major curvature after the first erect radiograph, but this did occur in some patients. The effect was likely dampened by the occurrence of TB. We observed T1–S1 height increases of 1.8 ± 1.8 cm, with 39% of patients (n = 14) experiencing a ≥ +1.5 cm T1–S1 height increase. It is possible for re-modelling of vertebra and disc to occur to some extent even after the growth spurt, accounting for our high level of clinical success.

Limitations of the current study include the lack of Sanders staging, as it is the most reported skeletal maturity indicator in the literature on VBT. The role of adjuvant therapies such as Schroth physical therapy in maintaining spinal alignment and preventing the need for revision surgery has not been evaluated here and should be the topic for future study. Clinical health-related quality of life (HRQOL) measures such as the SRS-22 and post-operative lumbar flexibility assessment would also be beneficial parameters to assess. Although HRQOL data were available for some patients, the majority did not have the questionnaires at the same time point; therefore, those data were not reported. Current VBT literature lacks uniform reporting of the HRQOL, a deficiency that needs to be rectified in future studies. We did not study lumbar flexibility, but this also should be the subject of future investigations. Additional work is required for more granular analysis comparing single- versus double-cord patients, clinically successful versus non successful patients, and outcomes based on skeletal maturity. Moreover, large, multi-center series with comparison to the standard of care spinal fusion would potentially provide more generalizable information and provide an important comparison to the standard approach.

## 5. Conclusions

In the largest single-surgeon series to date, with a minimum 2-year FU, we report on the outcomes of thoracolumbar VBT. We found reliable improvement of the major and minor curvatures without creating lumbar flatback. We also report a relatively low complication rate (10.8%) with all but one patient avoiding spinal fusion. Overall, 33 out of 37 patients (89%) were clinically successful.

## Figures and Tables

**Table 1:** Demographics of cohort treated with VBT.

Cohort Demographics (*N* = 37)	
Gender (F)	32 (86.5%)
Age (Years)	14.1 ± 1.6
Major Cobb (°)	51 ± 8 (38–71)
Major Cobb Fulcrum Bend Magnitude (°)	12 ± 10 (1–41)
Minor Cobb (°)	37 ± 13 (15–64)
Minor Cobb Fulcrum Bend Magnitude (°)	18 ± 11 (1–38)
Risser 0|1|2|3|4	4 (10.8%)|2 (5.5%)|5 (13.5%)|10 (27.0%|16 (43.2%)
PHOS * ⁺ 3A|3B|4	10 (27.8%)|8 (22.2%)|18 (50%)
Lenke 5|6	27 (73%)|10 (27%)
TRC (open)	2 (5.4%)
Instrumented Curve	
Thoracic and Thoracolumbar	15 (40.5%)
Thoracolumbar	22 (59.5%)
Mean Follow-up (months)	35.9 ± 11.5

⁺ 1 patient missing PHOS. * Proximal Humeral Ossification System (PHOS).

**Table 2:** Peri-operative characteristics of the cohort.

	TL/L Instrumented Curve (*N* = 22)	Both Curves Instrumented (*N* = 15)	*p* Values
Lenke 5|Lenke 6	21 (95%)|1 (5%)	6 (40%)|9 (60%)	0.0003
Lumbar Cords Used, 1 cord|2 cords	11 (50%)|11 (50%)	9 (60%)|6 (40%)	0.315
Operative Time (Min)	142.6 ± 37.5	236.4 ± 41.1	<0.0001
Estimated Blood Loss (cc)	126.3 ± 74.1	213.3 ± 142.3	0.0178
Disc Release	0	1 (7%)	-
Thoracoplasty	0	3 (20%)	-
LOS (Days)	4.0 ± 1.0	4.9 ± 1.3	0.022
Length of Chest Tube Placement (Days)	TL: 2.3 ± 0.6	T: 3.9 ± 1.3 TL: 2.7 ± 0.8	T: - TL: 0.0498

**Table 3:** Radiographic outcomes for patients with instrumented thoracolumbar/ lumbar curvatures.

	TL/L-Only Instrumented Curve (*N* = 22)				
	Pre	First Erect	Latest FU	*p*-Value *	*p*-Value **
Proximal Thoracic Curve (°)	8 ± 5	9 ± 6 (14 ± 21%)	8 ± 6 (11 ± 93%)	0.5098	0.3335
Thoracic Curve (°)	29 ± 8	17 ± 8 (44 ± 21%)	15 ± 11 (52 ± 42%)	0.5766	<0.0001
Lumbar Curve (°)	48 ± 6	16 ± 8 (66 ± 17%)	18 ± 12 (62 ± 27%)	0.1126	<0.0001
T5-T12 Kyphosis (°)	20 ± 10	21 ± 9	24 ± 14	0.6701	0.323
Lumbar Lordosis	53 ± 13	51 ± 9	52 ± 12	0.4664	0.6043
T1–S1 Height	41.2 ± 2.9	42.6 ± 2.8	44.4 ± 2.7	0.0004	0.0008

* Change in radiographic measurement from 1st erect to latest follow-up. ** Change in radiographic measurement from baseline to latest follow-up.

**Table 4:** Radiographic outcomes of patients with bilateral instrumentation.

	Both Curves Instrumented (*N* = 15)				
	Pre	First Erect	Latest FU	*p*-Value *	*p*-Value **
Proximal Thoracic Curve (°)	16 ± 6	12 ± 5 (24 ± 21%)	11 ± 7 (32 ± 44%)	0.3445	0.0026
Thoracic Curve (°)	49 ± 8	21 ± 8 (57 ± 15%)	20 ± 7 (59 ± 15%)	0.5112	<0.0001
Lumbar Curve (°)	56 ± 8	17 ± 7 (70 ± 13%)	22 ± 9 (60 ± 17%)	0.0128	<0.0001
T5-T12 Kyphosis (°)	26 ± 12	29 ± 16	32 ± 17	0.1416	0.0441
Lumbar Lordosis (°)	60 ± 14	53 ± 15	59 ± 15	0.0258	0.6435
T1–S1 Height	40.3 ± 3.6	42.9 ± 3.1	44.4 ± 3.4	0.0019	0.0002

* Change in radiographic measurement from 1st erect to latest follow-up. ** Change in radiographic measurement from baseline to latest follow-up.

## Data Availability

Data will be maintained for 7 years post publication of this study as required by local IRB regulations.
